# Elderly patients’ (≥65 years) experiences associated with discharge; Development, validity and reliability of the Discharge Care Experiences Survey

**DOI:** 10.1371/journal.pone.0206904

**Published:** 2018-11-07

**Authors:** Ranveig Marie Boge, Arvid Steinar Haugen, Roy Miodini Nilsen, Stig Harthug

**Affiliations:** 1 Department of Clinical Sciences, University of Bergen, Bergen, Norway; 2 Department of Medicine, Haukeland University Hospital, Bergen, Norway; 3 Department of Anaesthesia and Intensive Care, Haukeland University Hospital, Bergen, Norway; 4 Department of Research and Development, Haukeland University Hospital, Bergen, Norway; 5 Faculty of Health and Social Sciences, Western Norway University of Applied Sciences, Bergen, Norway; Imperial College, London, UNITED KINGDOM

## Abstract

**Background:**

A review of the literature reveals a lack of validated instruments that particularly measure quality in the hospital discharge process. This study aims to develop and validate a survey instrument feasible for measuring quality (≥65 years) related to the discharge process based on elderly patients’ experiences.

**Methods:**

Construction of the Discharge Care Patient Experience Survey (DICARES) was based on 16 items identified by literature reviews. Intraclass correlation for test–retest was applied to assess consistency of the survey. Explorative factors analysis was applied to identify and validate the factor structures of the DICARES. Cronbach’s α was used to assess internal reliability. To evaluate the external validity of the final DICARES questionnaire the patients’ scores were correlated with scores obtained from the three other questionnaires; the Nordic Patient Experiences Questionnaire, the 12-Item Short-Form Health Survey and Subjective Health Complaints. The DICARES association with readmissions was examined.

**Results:**

A total of 270 patients responded (64.4%). The mean age of participants was 77.1 years and 57.8% were men. The exploratory factor analysis resulted in a 10-item instrument consisting of three factors explaining 63.5% of the total variance. The Cronbach’s α were satisfactory (≥70). Overall intraclass correlation was 0.76. A moderate Spearman correlation (rho = 0.54, p <0.01) was found between the total mean DICARES score and total mean score of the Nordic Patient Experiences Questionnaire. The total mean DICARES score was inversely associated with the quality indicator based on readmissions (OR 0.62, CI 95: 0.41–0.95, p = 0.028)

**Conclusion:**

We have developed a 10-item questionnaire consisting of three factors which may be a feasible instrument for measuring quality of the discharge process in elderly patients. Further testing in a wider population should be carried out before implementation in health care settings.

## Background

To discharge elderly patients from hospitals or transfer these patients between different levels of health care institutions is one of the most significant challenges in health care services [1–4]. The discharge process includes a wide range of care professionals in a variety of settings, and it’s a coordinated, patient-centred, transparent process starting before admission if possible, or as soon after admission as suitable [5]. To recover and gain health after hospitalization adherence to therapy is crucial [6, 7]. According to the National Health Services (NHS), England, United Kingdom, people have a key role in protecting their own health, and to choose suited treatment and handle long-term conditions. Self-management/self-care is a term used to include all initiatives a person do to acknowledge, treat, and maintain his own health [7]. This may be done independently or in association with the healthcare system. The wellbeing and the safety in elderly patients are particularly at risk in the discharge process, because aging makes us more vulnerable due to loss of physical and mental function, and also the increased disease burden [8, 9]. Hence, quality in the discharge process requires caring interaction with the patients and their caregivers, and it involves considerable and accurate cooperation within the hospital as well as with the municipalities. Despite this knowledge, patients and caregivers are commonly unprepared for what will emerge after discharge, and they are frustrated by having to perform tasks their health care practitioners have left undone [10]. Introduction of experience surveys that provide feedback to the hospital departments can be one of several measures to improve the quality of the discharge process [1]. Even though readmission rate is nonspecific and may be affected by various conditions many healthcare organizations use it as an overall quality indicator [11]. There is a need for a more suitable approach to assess hospital performance in the patient discharge process. Patient experience; “*the sum of all interactions influenced by all interactions shaped by an organization’s culture across the continuum of care*” [12], has been recognized as an important facet of understanding quality since it reveals strengths and weaknesses in respect of efficiency and safety [12–14]. A survey about the experiences may be directed against specific parts of the care, not only as a global indicator.

Patient satisfaction and patient experiences are overlapping and both are important parts of healthcare quality [15]. The main difference between the concepts’ is that if patients are asked to rate how satisfied they are, the ratings tend to be very positive, while specific questions about the patients’ experiences with respect to certain processes provide more variation and are useful to customize interventions [16]. A systematic review by Doyle et al. [17] shows a positive association between examinations of patient experience, patient safety and clinical efficacy, and patient experience surveys may therefore pose as an appropriate basis of a quality indicator in general. Elderly patients express a clear preference to participate, but experience that the actual practice of involving old people in the discharge process is not well developed [18, 19]. Few studies have explored quality by use of patients’ experiences, and this deficiency of understanding the patient perspective has delayed the ability of hospitals to establish interventions which address these underlying causes of readmission [20]. Tools to measure patient experiences of quality of hospital care have been developed [1, 21], but the feasibility for use in quality improvement work is limited due to methodological weaknesses such as questionnaire design, patient selections, the data collection process, and data entry errors [22]. Patient experience surveys can be applicable as targeted tools of good scientific standards if performed by skilled scientists [22].

The discharge process must emphasize the patients’ ability to take care of themselves after hospitalization [7], tools for quality assessments in the discharge process should therefore cover questions about how patients’ experience the first period after hospitalization. These experiences may reflect the quality of the tasks performed by health care personnel during the discharge process. However, we have not been able to find such appropriate and validated instruments. This study aimed to develop a feasible brief survey instrument to identify elderly patients’ experiences with the hospital discharge process and with the following period after hospitalization, and to examine its reliability, internal validity, and to test the external validity.

## Methods

### Design

A cross–sectional study design was chosen to develop and validate the Discharge Care experience Survey (DICARES), http://dx.doi.org/10.17504/protocols.io.sm2ec8e.

In the planning phase of the study we discussed the object of the study with an established group of patient representatives at our hospital. Input from the group was included in the design of the questionnaire.

### Setting and study sample

Hospitals in Norway are owned by the state and the municipalities are committed to give health care services to their inhabitants when needed [3]. There is a written agreement between our hospital and the municipalities within the regional health authority. The municipality will be informed within 24 hours after admission if it is likely that the patient will need health care services from the municipality after discharge. The discharge planning start as soon after admission as possible for patients acutely admitted to hospital, and before admission for elective patients, if required. Elderly patients unable to take care of themselves after hospitalization are provided home based care services or nursing home facilities. Discharge planning includes assuring that the patients have got necessary aid equipment’s at home, and to inform the municipality or caregivers whether the patient is at risk of malnutrition, fall or pressure ulcers, with a plan for follow up when needed. Before leaving the hospital the patients will have an updated list of medication, a written patient information letter, have got a follow up appointment if required, and have had a discharge conversation with health care personnel responsible for the treatment. The patient’s general practitioner will receive a discharge letter from the hospital a week after discharge. To what degree health care personnel conform to these procedures is not documented.

In order to include elderly patients with significant comorbidities we recruited inpatients from five medical wards and one orthopaedic ward at a large tertiary teaching hospital in Bergen, Norway. Patients 65 years and above were included if hospitalized for more than 24 hours and were able to give their written informed consent. Patients living in nursing homes and patients with recognized reduced cognitive function were not included. Patients that met the inclusion criteria were invited to participate in the study by personal contact with the corresponding author. A paper-based survey including a pre-paid return envelope was sent to the patients approximately 30 days after hospital discharge during June 2013 to February 2015. Patients that did not respond within three weeks were reminded once by phone. Age and sex were recorded anonymously for non-responders. Data were plotted twice by two research assistants, and quality controlled for errors.

### Survey development

In order to develop the DICARES we conducted a systematic literature search (S1 File) during February and March 2013 in the databases Medline, Embase, Cinahl, SveMed and PsycINFO. We adapted several elements from the PRISMA checklist as guidance. None of the databases used *patient experience* as MeSH term, therefore we applied *patient satisfaction* and *patient perspective*, and further *patient discharge*, *patient transfer*, *continuity of patient care*, *patient hand over*, *patient hand off*, *primary health care*, *home based care*, *nursing homes*, *community health services and*, *community based care*. The literature review identified 736 matches, reduced to 528 after duplicate control. Twenty–four abstracts met the inclusion criteria; qualitative and quantitative studies in English, Norwegian, Danish or Swedish, aimed at patients of both sex ≥ 65 years with hospital stays in somatic departments. If there were other participant groups in addition to the patients, for example relatives and / or healthcare personnel, it had to be clear what the patient’s experience was. Research before year 2000 had to be available both as an abstract and in full electronic text to be included. Intervention, follow-up and evaluation studies were excluded. Relevant matches were read in full text, and eight articles filled the criteria. Further we did a literature review in order to find questionnaires that included questions concerning the discharge process and the following weeks after hospitalization. To include candidate items for the new DICARES questionnaire based on the literature reviews we used an eclectic approach. An expert panel evaluated if the items were relevant to be included in the questionnaire. The expert panel consisted of researchers, health care personnel, and leaders at the hospital. Three items regarding patient participation were derived from a 15-item validated survey developed by Coleman et.al. [2] designed to be used by patients 18 years and older. The complete 15-item survey does not provide substantial more information than the three core questions selected [23]. Two more items on patient participation were obtained from a study of elderly patients by Foss et.al. [18]. Further, we included eleven items based on a 36-item survey developed by Kangovi et.al. [20]. These items are related to daily lives activities, adherence to discharge medications and emotional problems following the period after hospitalization from readmitted patients’ perspective. A total of 16 candidate items were translated and adjusted to fit our setting with respect to language, design, formatting and methodology (Table 1).

**Table 1:** Sixteen items were identified in the literature to be included in the new questionnaire.

Item number	Original phrasing	Adjusted phrasing
1	I got the opportunity to tell the staff what i myself considered important in order to manage after discharge ᵇ	In connection with being discharged, I had an opportunity to notify hospital personnel about what I thought was important ᵇ
2	The hospital staff took my preferences and those of my family or caregiver into account in deciding what my health care needs would be when I left the hospital ᶜ	The hospital staff took into account the wants and needs of both myself and my relatives in deciding which healthcare services I would need when I was discharged from hospital ᶜ^,^ ᵈ
3	When I left the hospital, I had a good understanding of the things I was responsible for in managing my health ᶜ	When I was discharged from hospital, I had a good understanding of my responsibility in terms of looking after my health ᶜ
4	When I left the hospital, I clearly understood the purpose of taking each of my medication ᶜ	When I was discharged from hospital, I understood thoroughly the purpose of taking my medication ᶜ
5	How important was it to you to influence the time of discharge? ᵇ	It was important for me to be able to influence when I was to be discharged from hospital ᵇ^,^ ᵈ
6	Did you feel like you needed to stay a bit longer the first time you were admitted, and you were discharged too early? ᵃ	I felt I was discharged too earlyᵃ
7	Did you have trouble understanding the discharge instructions? ᵃ	I have experienced problems in understanding the instructions I received when I was discharged from hospital ᵃ
8	Did you have trouble following the discharge instructions? ᵃ	I have had problems in following the instructions I received when discharged from the hospitalᵃ
9	Did you have trouble getting help from your outpatient doctor? ᵃ	I have experienced problems receiving help from my GP ᵃ^,^ ᵈ
10	Did you have trouble with taking your medications? ᵃ	I have experienced problems taking my medicines ᵃ^,^ ᵈ
11	Did you have trouble getting your medications after you last left the hospital? ᵃ	I have experienced problems getting hold of medicines ᵃ^,^ ᵈ
12	Did you have trouble with your daily activities since you last left the hospital, for example bathing, **eating**, and using the bathroom? ᵃ	I have experienced problems in getting sufficient nutrition ᵃ
13	Did you have trouble with your daily activities since you last left the hospital, for example bathing, eating, and using the bathroom? ᵃ	I have experienced problems in performing daily activities (e.g. personal hygiene, getting dressed or cooking) ᵃ
14	Did you struggle with stress or depression? ᵃ	I have felt stressed ᵃ
15	Similar to item number 14 ᵃ	I have been depressed ᵃ
16	Did you wish you had more people to talk to and give you moral support after you got home from the hospital? ᵇ	I wish I had more people to talk to, to support me following discharge from hospital ᵇ^,^ ᵈ

The 16 statements were based on items retrieved from the following studies

ᵃ Kangovi et al.2012

ᵇ Foss et al.2011, and

ᶜ Coleman et al. 2005

ᵈ Item not included in the final DICARES questionnaire

Forward translation of the DICARES was performed by two Norwegian registered nurses / researchers with knowledge of English language. Backward translations were completed by two independent native English translators with no prior knowledge of the questionnaire [24]. Inadequate expressions or concepts of the translation were discussed within the bilingual expert panel. All items were scored on a five point Likert-like scale: *Not at all*, *To a little extent*, *To some extent*, *To a large extent and To a very large extent*, and assigned values 1, 2, 3, 4 and 5, respectively. Values from negative statements (number 6, 7, 8, 9, 10, 11, 12, 13, 14, 15 and 16) were inverted to positive values. High score means a better condition for the patient. The total score was calculated as the mean of the items’ scores.

Our survey consisted of four questionnaires, with a total of 66 questions. This included the 16-item DICARES-candidate and three validated questionnaires: the eight-item Nordic Patient Experiences Questionnaire (NORPEQ) measuring quality of care in general in Norwegian hospitals based on six validated items [25, 26], the 12-Item Short-Form Health Survey (SF-12) assessing general health status (Physical Composite Scale and Mental Composite Scale) [27], and the 29-item symptom specific questionnaire Subjective Health Complaints (Musculoskeletal pain, Pseudoneurology, Gastrointestinal problems, Allergies and Flu) (SHC) [28, 29]. Additional one question about readmission within 30 days was included. Readmission data within 30 days [30] was also recorded from the electronically patient administrative system. Further information from this system included age, sex, date of admission, length of stay, International Classification of Diseases-10th version (ICD-10) codes [31], and a calculated Charlson comorbidity index [32] based on the ICD-10 codes.

### Survey validation and statistics

When planning the study to explore the psychometric properties sample size was calculated to be at least 250 for detecting differences in the total DICARES score with outcome readmission, expected exposure 20%, power 80% and p<0.05. Population mean score values for each item were imputed if eight or more items scores in the initial 16 -item DICARES were completed. To investigate face validity [33] the first 22 patients who returned the survey, and had completed the 16 initial DICARES items, answered five additional identical questions, assessing whether the items were understandable, meaningful, relevant, easy to answer, and whether they were too personal. Nineteen patients returned the evaluation form, and based on the findings no items required to be changed. In order to assess test-retest reliability, fifty respondents were asked to complete the DICARES a second time after 21 days. Intraclass correlation (ICC) between the items was examined for consistencies in the test re-test measure. ICC estimates and their 95% confidence intervals (CI) were calculated based on a mean-rating (k = 2), absolute-agreement, two-way mixed-effects model [34], and was estimated using the following interpretation: Poor (0.40> r), Fair (0.59> r >0.40), Good (0.74> r >0.60 and Excellent (1.00> r >0.75) [35]. Explorative factor analysis was applied to identify the factor structure of the DICARES questionnaire as described by Pett et al. [36]. To assess the sampling adequacy, we used the Kaiser-Meyer-Olkin measure, and the Bartlett’s test of sphericity [36]. Principal component analysis with Varimax rotation was applied to identify factors structures of the initial 16 candidate items measuring patient experiences. Eigenvalues >1 was used to identify the number of factors, and absolute value for factor loadings was ≥0.30. If an item loaded moderate or strong to more than one factor, the item was allocated to the factor where it got the highest loading. The item with the highest loading was placed first in the factor.

Inter-correlation between the items of each factor was examined for internal reliability using Cronbachs’α and values ≥0.7 were considered acceptable [37]. Construct validity was assessed by inter-correlation of the factors, and by correlation of the final total DICARES score to the NORPEQ, the SF-12 and the SHC, using Spearman’s correlation coefficient. Moderate correlation coefficients between 0.30–0.49 were considered satisfactory [38]. Further, for external validity mean score of the final total DICARES score was compared with the mean NORPEQ score for the readmitted and non-readmitted patients, and tested using a two-sample t-test. For comparison with the previously validated NORPEQ questionnaire the total DICARES score was transformed to a 0–100 scale by subtracting one from the mean score of each item and then multiplying with 25. The external validation comprised a multiple stepwise, backward logistic regression analysis including DICARES total score, Charlson Comorbidity Index, sex, and age as independent variables and readmission within 30 days as a binary dependent variable. Missing data in the regression analysis were handled using complete case analysis. Statistical analyses were performed with SPSS version 23.0 (IBM Corp., Armonk, NY). All p-values were two-sided, and values <0.05 were considered statistically significant.

### Ethics

#### Ethics approval and consent to participate

This study was conducted in accordance with the Helsinki Declaration [39], and was accepted by the Western Norway Regional Committee for Medical and Health Research Ethics (Ref.: 2013-401b). The study was also approved by the respective hospital managers. Patients involved in the process signed a consent form prior to hospital discharge. Data from the survey, and questionnaires, were stored in a designated server at the hospital.

## Results

### Study sample

**Fig 1:**
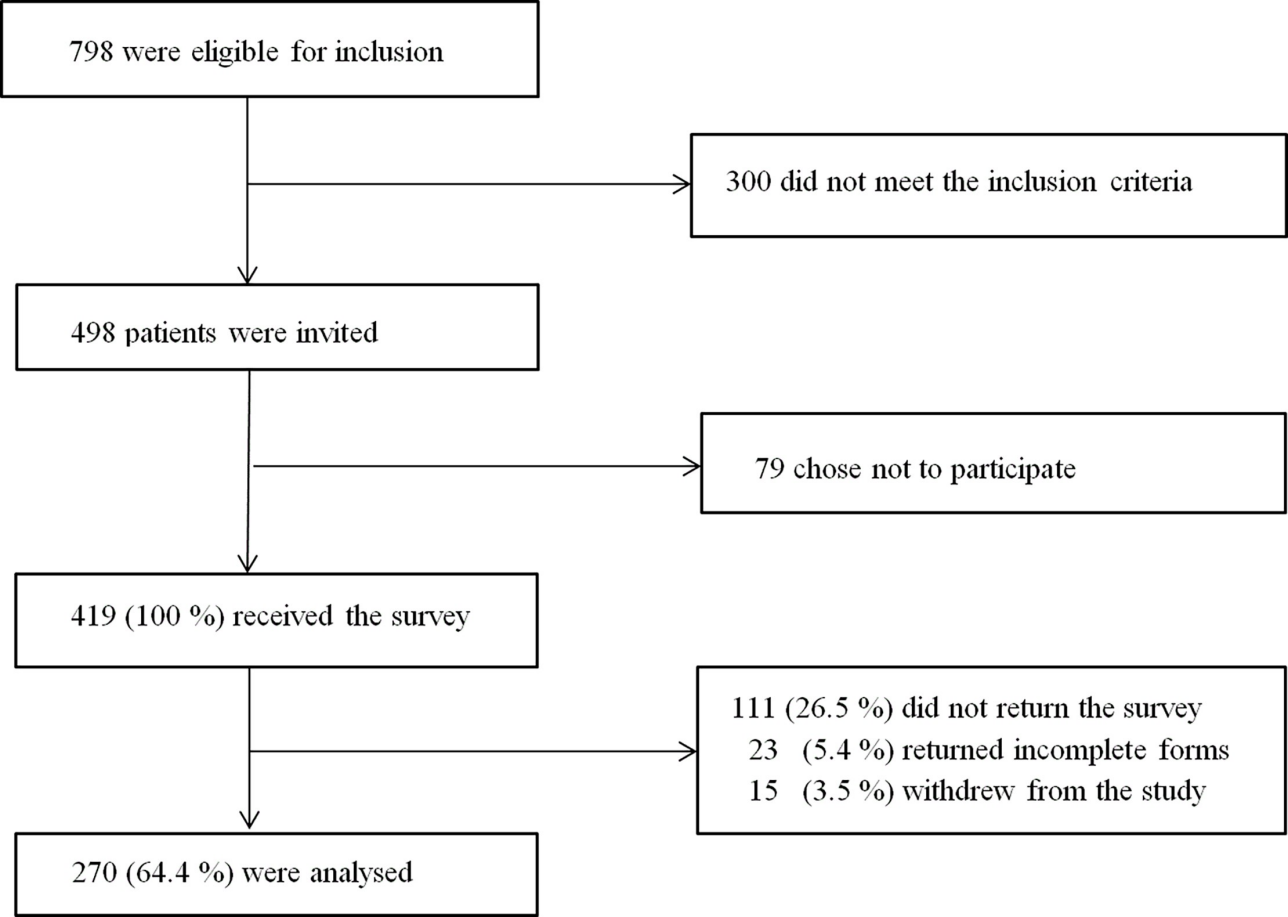
Flow chart of patient inclusion in the study. Elderly inpatients (≥65) were recruited from five medical wards and one orthopaedic ward at Haukeland University Hospital, Bergen, Norway.

A flow chart of the recruitment to the study is shown in Fig 1. Initially, a total of 798 patients were eligible for inclusion and 419 of the 498 patients who met the inclusion criteria consented to participate. Of these 270 returned completed questionnaires, yielding a response rate of 64.4%.

#### Patient characteristics

The mean age of participants was 77.1 year (SD 7.2; range 65–98), and men accounted 57.8% of the sample (Table 2). The mean length of hospital stay was 8.3 days (SD 8.8; range 2–80; median 6.0) and 90% of the patients were discharged from medical wards. Fifty-one percent of the patients had more than three diagnoses, and the mean Charlson Comorbidity Index was 1.61 (SD 1.5). Seventy-two patients (26.7%) were readmitted to hospital within 30 days after discharge. The mean age for non-responders (n = 228) was 78.8 years (range: 65–94), and did not deviate significantly from the responders. There were significantly fewer men among non-responders than responders (34.4% versus 57.8%, p <0.001).

**Table 2:** Characteristics of the participants included from Haukeland University Hospital, Bergen, Norway.

Variables	n (%)
All patients	270 (100)
**Age groups (years), mean (SD)**	77.1 (7.2)
• 65–75	127 (47.0)
• 76–85	99 (36.7)
• 86–98	44 (16.3)
**Sex**	
• Women	114 (42.2)
• Men	156 (57.8)
**Marital status**	
• Married or cohabitant	165 (62.5)
• Not married/cohabitant	14 (5.2)
• Widow /widower	66 (24.4)
• Separated/divorced	19 (7.0)
• Not answered	6 (2.2)
**Length of in-hospital stay by days, mean (SD)**	8.3 (8.8)
**Departments**	
• Medical departments ᵃ	243 (90.0)
• Orthopaedic department	27 (10.0)
**Readmitted**	72 (26.7)
**Charlson Comorbidity index, mean (SD)**	1.61(1.5)
• 0 points	60 (22.2)
• 1 point	100 (37.0)
• ≥ 2 points	110 (40.7)

ᵃ Department of Pulmonary Diseases, Department of Heart Diseases, and Department of Medicine.

### The DICARES scores

Nineteen questionnaires had less than 50% of the 16 items completed. The completion rate of the individual 16 items varied from 79.6% to 99.3% (Table 3). The total mean score for the 16 candidate items was 4.06 (SD 0.57). The lowest score was observed for item *Opportunity to notify what was important* (2.93, SD 1.13), whereas the highest score was observed for item *Problems taking medicines* (4.78, SD 0.67). Forty-five (90%) of the 50 re-test questionnaires were returned. The overall ICC was Excellent (0.76, CI 95; 0.70, 0.82), results for single-item measures are shown in Table 4.

**Table 3:** Item scores of the initial 16-item Discharge Care Experience Survey (DICARES) (n = 270).

		Respondents	Number of scores of the five point scale (%)										
	Items (abbreviated)	n (%)	1		2		3		4		5		Mean (SD)
1	Opportunity to notify what was important	222 (82.2)	33	(12.2)	56	(20.7)	51	(18.9)	58	(21.5)	24	(8.9)	2.93 (1.13)
2	The hospital staff took into account wants and needs ᵃ	215 (79.6)	31	(11.5)	29	(10.7)	39	(14.4)	75	(27.8)	41	(15.2)	3.31 (1.18)
3	Understanding of health responsibility	252 (93.3)	11	(4.1)	14	(5.2)	43	(15.9)	129	(47.8)	55	(20.4)	3.81 (0.95)
4	Understanding the purpose of medication	259 (95.9)	4	(1.5)	14	(5.2)	22	(8.1)	104	(38.5)	115	(42.6)	4.20 (0.90)
5	Influence when to be discharged ᵃ	237 (87.8)	28	(10.4)	36	(13.3)	66	24.4)	78	(28.9)	29	(10.7)	3.19 (1.11)
6	Discharged too early ᵇ	262 (97.0)	145	(53.7)	42	(15.6)	42	(14.5)	16	(5.9)	17	6.3)	4.08 (1.22)
7	Problems in understanding the instructions ᵇ	249 (92.2)	158	(58.5)	57	(21.1)	23	(8.5)	6	(2.2)	5	(1.9)	4.43 (0.86)
8	Problems in following the instructions ᵇ	250 (92.6)	157	(58.1)	62	(23.0)	22	(8.1)	7	(2.6)	2	(0.7)	4.43 (0.86)
9	Problems receiving help from GP ᵃ^,^ ᵇ	244 (90.4)	179	(66.3)	33	(12.2)	13	(4.8)	12	(4.4)	7	(2.6)	4.50 (0.95)
10	Problems taking medicines ᵃ^,^ ᵇ	262 (97.0)	229	(84.8)	17	(6.3)	8	(3.0)	17	(6.3)	2	(0.7)	4.78 (0.67)
11	Problems getting hold of medicines ᵃ^,^ ᵇ	259 (95.9)	219	(81.1)	20	(7.4)	12	(4.4)	5	(1.9)	3	(1.1)	4.73 (0.73)
12	Problems in getting sufficient nutrition ᵇ	265 (98.1)	149	(55.2)	49	(18.1)	48	(17.8)	11	(4.1)	8	(3.0)	4.21 (1.06)
13	Problems in performing daily activities ᵇ	267 (98.9)	108	(40.0)	69	(25.6)	51	(18.9)	27	(10.0)	12	(4.4)	3.88 (1.17)
14	Felt stressed ᵇ	263 (97.4)	119	(44.1)	77	(28.5)	46	(17.0)	15	(5.6)	6	(2.2)	4.10 (1.01)
15	Been depressed ᵇ	268 (99.3)	142	(52.6)	51	(18.9)	56	(20.7)	12	(4.4)	7	(2.6)	4.15 (1.06)
16	More people to talk to ᵃ^,^ ᵇ	254 (94.1)	103	(38.1)	62	(23.0)	45	(16.7)	30	(11.1)	14	(5.2)	3.83 (1.20)
Total mean score 16 items		270ᶜ (100)											4.06 (0.57)

ᵃ Not included in the final DICARES model

ᵇ Negative statements were inverted to a positive scale

ᶜ Population mean score values for each item were imputed if eight or more items were completed.

**Table 4:** Test-retest of the initial 16-item Discharge Care Experience Survey (DICARES).

		Item scores 30 days after discharge		Item scores 51 days after discharge		* *
	Items (abbreviated)	n	Score (SD)	n	Score (SD)	Intraclass correlation (95% CI)
1	Opportunity to notify what was important	34	3.0 (1.1)	37	2.8 (1.1)	0.56 (0.26, 0.76)
2	The hospital staff took into account wants and needs ᵃ	34	3.8 (1.3)	35	3.3 (1.3)	0.76 (0.57, 0.88)
3	Understanding of health responsibility	44	3.9 (0.8)	44	4.0 (0.8)	0.60 (0.36, 0.76)
4	Understanding the purpose of medication	45	4.3 (1.0)	44	4.2 (0.9)	0.69 (0.49, 0.82)
5	Influence when to be discharged ᵃ	38	3.0 (1.1)	40	3.0 (1.1)	0.65 (0.41, 0.81)
6	Discharged too early ᵇ	44	4.6 (0.7)	44	4.2 (1.2)	0.63 (0.40, 0.78)
7	Problems in understanding the instructions ᵇ	43	4.6 (0.7)	43	4.5 (0.7)	0.48 (0.21, 0.68)
8	Problems in following the instructions ᵇ	43	4.5 (0.9)	43	4.6 (0.6)	0.42 (0.13, 0.64)
9	Problems receiving help from GP ᵃ^,^ ᵇ	44	4.9 (0.4)	43	4.6 (1.0)	0.65 (0.43, 0.79)
10	Problems taking medicines ᵃ^,^ᵇ	44	4.8 (0.5)	45	4.8 (0.5)	0.52 (0.27, 0.71)
11	Problems getting hold of medicines ᵃ^,^ ᵇ	44	4.4 (0.9)	44	4.8 (0.5)	0.86 (0.75, 0.92)
12	Problems in getting sufficient nutrition ᵇ	45	4.1 (1.0)	45	4.2 (1.0)	0.76 (0.61, 0.86)
13	Problems in performing daily activities ᵇ	45	4.3 (1.0)	44	4.2 (1.0)	0.61 (0.39, 0.77)
14	Felt stressed ᵇ	45	4.3 (1.0)	45	4.2 (1.1)	0.73 (0.55, 0.84)
15	Been depressed ᵇ	43	3.9 (1.3)	44	4.2 (1.1)	0.81 (0.68, 0.90)
16	More people to talk to ᵃ^,^ ᵇ	43	3.9 (1.3)	44	3.8 (1.1)	0.73 (0.55, 0.84)

ᵃ Item not included in the final DICARES questionnaire

ᵇ Negative statements were inverted to a positive scale.

#### Extracting factors from items

Results from the exploratory factor analyses are shown in Table 5. The Kaiser-Meyer-Olkin measure of sampling adequacy was estimated to be 0.75, whereas the p-value for the Bartlett’s test of sphericity was <0.001.The estimated communalities varied between 0.4 and 0.8. Eigenvalues were 3.65, 1.63 and 1.06 for the three factors, and a total of 10 items were included in the final DICARES-model, explaining 63.5% of the common variance. All 10 items loaded satisfactorily (range of factor loadings 0.50–0.91) in the rotated component matrix. The three factors were named: *Coping after discharge* (three items), *Participation in discharge planning* (three items) and *Adherence to treatment* (four items). The corresponding Cronbach’s α for internal reliability were 0.73, 0.71 and 0.70, respectively. A moderate relationship between the three factors ranged from a Spearman’s correlation coefficient for internal validity was 0.32 to 0.47 (p = 0.01). The DICARES total mean score (10 items) was 4.04 (SD 0.65).

**Table 5:** Factors of the Discharge Care Experience Survey (DICARES) according to explorative factor analysis.

		Explorative Factor Analysis ᵃ	
Factors	Item/ total correlation	Loading	Cronbach's α
**Coping after discharge**			0.73
1.I have felt stressed ᵇ	0.47	0.91	
2. I have been depressed ᵇ	0.55	0.86	
3. I felt I was discharged too early ᵇ	0.43	0.50	
**Participation in discharge planning**			0.71
4. When I was discharged from hospital, I had a good understanding of my responsibility in terms of looking after my health	0.39	0.84	
5.When I was discharged from hospital, I under-stood thoroughly the purpose of taking my medication	0.45	0.76	
6. In connection with being discharged, I had an opportunity to notify hospital personnel about what I thought was important	0.32	0.72	
**Adherence to treatment**			0.70
7. I have experienced problems in getting sufficient nutritionᵇ	0.31	0.77	
8. I have had problems in following the instructions I received when discharged from the hospital ᵇ	0.62	0.68	
9. I have experienced problems in performing daily activities (e.g. personal hygiene, getting dressed or cooking) ᵇ	0.56	0.64	
10. I have experienced problems in understanding the instructions I received when I was discharged from hospital ᵇ	0.59	0.64	
Item—total correlation			0.79

Table footnote:

ᵃ Rotated Component Matrix. Extraction Method: Principal Component Analysis. Rotation Method: Varimax with Kaiser Normalization

ᵇ Negative statements were inverted to a positive scale.

#### Comparison of the DICARES to validated instruments, age and comorbidity

The DICARES total score correlated moderate positively to the NORPEQ score (Spearman’s rho = 0.54), SF-12 Mental Composite Scale score (Spearman’s rho = 0.55), and inversely with SHC Pseudoneurology (Spearman’s rho = -0.47), and SHC Musculoskeletal (Spearman’s rho = -0.36) (Table 6). Patients readmitted within 30 days scored significantly lower to the DICARES than those not readmitted (Table 7). Equivalent results were not recognized for the NORPEQ. As shown in Table 8, the DICARES and the Charlson Comorbidity Index were significantly associated with readmission.

**Table 6:** Correlation between The Discharge Care Experience Survey (DICARES) and relevant measurements (n = 270).

Questionnaires	Spearman's rho
The Nordic Patient Experiences Questionnaire (NORPEQ) (n = 266)	0.54 ᵃ
The 12 Item Short Form Survey (SF-12) (n = 269)	
• Physical Composite Scale	ᵃ
• Mental Composite Scale	0.55 ᵃ
The Subjective Health Complaints (SHC) (n = 250)	
• Musculoskeletal pain	-0.36 ᵃ
• Pseudoneurology	-0.47 ᵃ
• Gastrointestinal problems	-0.26 ᵃ
• Allergies	-0.29ᵃ
• Flu	-0.13 ᵇ

ᵃ Spearman's correlation is significant at the 0.01 level (2-tailed)

ᵇ Spearman's correlation is significant at the 0.05 level (2-tailed).

**Table 7:** Comparison of the DICARES ᵃ and the NORPEQ ᵇ to 30 days readmission.

		Not readmitted		Readmitted ᵈ	
	n	Mean % (SD)	n	Mean % (SD)	P-value ᵉ
The DICARES ᶜ	198	77.5 (15.4)	72	72.0 (17.8)	0.014
The NORPEQ	196	75.2 (14.8)	70	75.0 (14.8)	0.930

ᵃ The Discharge Care Experience Survey

ᵇ The Nordic Patient Experiences Questionnaire

ᶜ The DICARES factors were converted to a scale from 0 to 100, where 100 is the best possible experience of care

ᵈ Patient journal data and patient reported data from the DICARES

^e^ Two-sample t-test.

**Table 8:** Logistic regression analyses of factors correlated to readmission (n = 270) ᵃ.

	Unadjusted model				Final adjusted model			
Independent variables	OR	95% CI		P-value	OR	95% CI		P-value
The DICARES ᵇ	0.60	0.40	0.91	0.015	0.62	0.41	0.95	0.028
Charlson Comorbidity Index	1.32	1.11	1.57	0.002	1.31	1.10	1.55	0.003
Sex	1.13	0.66	1.95	0.656				
Age	1.03	0.49	2.17	0.939				

ᵃ Number of valid responses

ᵇ The Discharge Care Experiences Survey.

## Discussion

In this study we have developed a questionnaire instrument for use in quality improvement work. The DICARES is a three factor questionnaire measuring patient experiences based on 10 items concerning discharge from hospital, and the following period after hospitalization. The DICARES showed moderate correlation to the validated quality survey instrument NORPEQ. In contrast to NORPEQ, low scores on the DICARES were associated to readmission.

Birkelien and Madison have developed a framework for improving quality by use of patient experience in hospitals [40]. The DICARES may be a useful tool to measure and monitor targeted interventions emanating from the multifaceted components suggested in this framework. We did not find validated patient experiences instruments that explicit measured quality in the discharge process. However, Bettie et al. identified 11 instruments in a systematic review of instruments to measure patient experience of healthcare quality in hospitals [1]. Three instruments from Hong Kong, Ethiopia and India respectively are not discussed in the present study for consideration of possible cultural differences. Four of the eight remaining instruments included questions related to the discharge process and/or transition; The NHS Inpatient Survey (NHSIP), the Scottish Inpatient Patient Experience Survey (SIPES), the Picker Patient Experience Questionnaire (PPE-15) and the Hospital Consumer Assessment of Healthcare Providers and Systems (HCAHPS). However, differences in approach, methodology and timing of administration limited comparison with the DICARS. In contrast to Kangovi et al. [20] we could not find that these four instruments included questions regarding self-care during the first period after the hospital stay. The SIPES got yes/no and three or more response alternatives, with timing of administration between four and five months after discharge, The PPE-15 got the same response alternatives whereas time of administration was within a month. The HCAHPS included the three Coleman’s items similar to the DICARES. Mode of administration of the HCAHPS was by mail, telephone, and interactive voice recognition, where data were collected between 48 hours to six weeks, and the instrument got a four- point Likert like scale. The Consumer Quality Index (CQI) questionnaire is based on the HCAHPS and was used by Smirnova et al. [41] in a study from 2017 that included almost 23,000 patients and where nearly half of the respondents were 65 years or older. The results showed that variations in the measurement of patient experiences could be attributed to variation in quality of care. Five items in the DICARES cover communication between the patients and the health care personnel, who has emerged as more important than previously thought [41]. The total mean score of the DICARES was 4.04, and somewhat higher than the score on the subscale *Information at discharge* according to the study of Smirnova et al. [41] with mean score 0.7, corresponding to 77% and 70% of maximum scores, respectively. Even if the results in the study of Smirnova et al. may be hampered by relatively low response rate and by methodological issues discussed by Felix et al. [22], we found this comprehensive study appropriate to compare with the DICARES, attributable to the large group of eldery patiets included in the study. The NORPEQ was included in the present study for comparison since it is used as a quality measurement in Norwegian hospitals [26]. Similar to the DICARES, the NORPEQ has statements scored on a five point Likert like scale and applies explorative factor analyses, which strengthen the credibility of comparison of the instruments.

The DICARES differed significantly in scores with respect to readmitted patients. This finding is in contrast to Felix et al. [22], who found results of the post discharge questionnaires were not associated with readmission. However, our finding regarding the DICARES, as compared to NORPEQ and readmission, is consistent to findings reported by Felix et al. [22]. In the development of the DICARES we have implemented some of the recommendations such as graded response scale instead of yes / no questions, which partly could explain the fact that we succeeded in identifying patients at risk of readmission. Another explanation for this result may be that DICARES covers statements related to self-care the first four weeks after discharge.

The DICARES items correspond with quality in the discharge process, for instance drug errors at the time of discharge can be a consequence of incomplete or inaccurate information, and as such, are important issues to survey [42]. The lowest response rate scores were found for the item *Opportunity to notify what was important*. This might reflect that older patients may encompasses rejection of own need, and are grateful and humble to the systems of care despite the lack of information and participation in the transition process [43]. It is less associated with instruments measuring health status (SF-12), or subjective health complaints (SHC). This is an argument for the DICARES as a questionnaire instrument reflecting quality in the discharge process. After adjusting for age and sex, a significant association was found between the DICARES, the Charlson Comorbidity Index and readmission, not unexpectedly the DICARES and the Charlson Comorbidity Index were independent determinants.

Factor analyses were performed to explore if the items included would give meaningful input in terms of understanding shortfalls in the discharge process, and to investigate whether the DICARES could provide hospitals with a tool for monitoring improvement processes. Validity testing of the instrument was considered satisfactory, and three factors were classified as acceptable. Naming of the factors; *Coping after discharge*, *Participation in discharge planning and Adherence to treatment* were suggestive as to what dimension each factor represents [36]. Participation in discharge planning has been recognized as important, even for very old patients [18], and it is advantageous at all levels in healthcare to empower patients and to improve services and health outcomes [44].

Coping is, according to Lazarus and Folkman, defined as *“constantly changing cognitive and behavioural efforts to manage specific external and/or internal demands that are appraised as taxing or exceeding resources of the person”* [45]. Comorbidity affects the relationship between coping and stress [4] and increased comorbidity is associated with higher severity levels of both depression and generalized anxiety [8]. Adherence require the patient’s agreement to recommendation, and is defined as “*the extent to which a person’s behaviour–taking medication*, *following a diet*, *and/or executing lifestyle changes-corresponds with agreed recommendations from a healthcare provider”* [46]. The World Health Organization claims that improving adherence to therapy would provide a significant advantage on investment through primary prevention [6].

A limitation is that our study was performed in one hospital on elderly patients predominantly from different medical departments, and one might argue that the study group is somewhat homogenous. However, the group included a satisfactory range of ages, sex and different medical conditions, and the final DICARES was acceptable with respect to missing data. We have no explanation for the relatively higher proportion of men among the responders than non-responders. The mean hospital stay was approximately eight days, which is somewhat higher compared to other studies [2, 4, 20, 25]. As compared to the national mean for all hospitalised patients, the readmission rate was above double. Higher age, and significant disease burden, may explain these findings [47]. After ethical considerations we decided to not include patients with reduced cognitive capacity, which also may represent a weakness in this study. These differences in population characteristics may influence the results, and limit the comparison with other studies. On the other hand, the study is performed in a well-defined patient population, and with careful sampling of data. The corresponding authors’ meeting with each patient, and that the consent form had to be signed before the patients’ were discharged probably contributed to the high response rate [48]. A limitation is that of the eligible 798 patients only 498 (38%) met the inclusion criteria and the most vulnerable patients were therefore not included in this study. To monitor and improve the discharge quality of patients not able to give informed consent, other methods than self–completing questionnaires should be employed. We do not know the reason for non-response and there is a potential for selection bias among the respondents even with the relatively high response rate.

A challenge with surveys performed after discharge is that many, especially frail elderly, might have a problem in remembering events four to five months after a hospital stay as used by some studies [1]. We chose a shorter time lag as this was relevant due to comparison with the quality indicator readmission within 30 days. It is possible that the moment that some respondents were asked to fill out the DICARES questionnaire coincided with their readmission. This might have negatively biased their perceptions of their previous hospitalization producing a recall bias. We chose to develop and validate a questionnaire on elderly patients’ experiences. This may be a weakness with respect to the fact that younger patients may have the same issues [49]. However, the largest numbers of patients on medical wards are elderly and they are also in particular at risk of unplanned hospital readmission ascribable to morbidity and functional decline [50]. Coleman’s three items obtained from the 15-item Care Transition Measure has the limitation that it was derived from a small single health plan in the USA and might over-represent behaviours specific to that plan, or the patients’ selection may be biased. They may not represent local European problems at discharge, which may be county / hospital / unit/ condition specific [51]. The psychometric properties of the DICARES were considered satisfactory.

## Conclusions

This hospital based study suggests that the DICARES may be a feasible questionnaire instrument for measuring quality based on experiences of the discharge process among elderly patients (≥65 years). Our study also indicates that the DICARES is capable of monitoring the quality of care of important issues concerning the discharge process, and can be used as an additional tool for quality improvement care processes in hospitals. To further develop this instrument, it needs to be tested in a larger sample with a broader representation of patients in different hospital departments. The three-factor structure should be confirmed using a confirmatory factor analysis.

## Supporting information

S1 File Search strategy. Systematic literature search of patients’ experiences transition from hospital to community health services.
(PDF)

S2 File Available data. Anonymous data set including 270 respondents.
(XLSX)

S3 File The Norwegian version of the initial 16 item DICARES questionnaire. Erfaringer knyttet til utskriving og tiden etter sykehusoppholdet.
(PDF)
