# Supplementary material for: Elderly patients’ (≥65 years) experiences associated with discharge; Development, validity and reliability of the Discharge Care Experiences Survey
Source: PLoS One. 2018 Nov 7;13(11):e0206904. doi: 10.1371/journal.pone.0206904 (PMC6221326; doi:10.1371/journal.pone.0206904)
Supplement: S1 File — Systematic literature search of patients’ experiences transition from hospital to community health services. (PDF) [file pone.0206904.s001.pdf]

Systematic_literature_search_Search_strategy.pdf

Flow_chart_systematic_literature_search.pdf

Results_Systematic_literature_search.pdf

Documentation of a systematic literature search in data bases between 20.2.13- 26.3.13

**PICO**	P: Elderly patients 65 years or older transferred from hospital to community health services , I: No intervention, C: No comparison, O: Patients' experience
**Title**	Elderly patients' (65 years or older) experiences with transition from hospital to community health services
**Librarian who supervised the research:**	Regina Küfner Lein

**Database/ resource:**	Ovid MEDLINE(R) In-Process & Other Non-Indexed Citations and Ovid MEDLINE(R) <1946 to Present>
**Date of search:**	20.2.2013
**Search history:**	1 exp Patient Discharge/ (17105)<br>2 exp Patient Transfer/ (5321)<br>3 "Continuity of Patient Care"/ (13306)<br>4 (hospital adj3 discharge).mp. or (patient* adj3 (transition or transfer or discharge)).tw. [mp=title, abstract,<br>original title, name of substance word, subject heading word, keyword heading word, protocol supplementary concept, rare<br>disease supplementary concept, unique identifier] (32015)<br>5 1 or 2 or 3 or 4 (59489)<br>6 limit 5 to "all aged (65 and over)" (19413)<br>7 exp Patient Satisfaction/ (54825)<br>8 (patient adj2 (perspective or experience*)).tw. (11165)<br>9 7 or 8 (65108)<br>10 6 and 9 (800)<br>11 Primary Health Care/ (49277)<br>12 exp Community Health Services/ (462391)<br>13 (primary adj2 (care or "health care" or healthcare)).tw. (76869)<br>14 (("home based" or "homebased" or "home-based" or home or short term or short-term or shortterm) adj2 care).tw.<br>(17792)<br>15 exp Nursing Homes/ (30411)<br>16 "nursing home*".tw. (20399)<br>17 11 or 12 or 13 or 14 or 15 or 16 (574562)<br>18 10 and 17 (336)<br>19 exp Patient Handoff/ (21)
**Number of matches:**	336 + 21 (4 of the 21 were perhaps relevante)

**Database /resource:**	Ovid MEDLINE(R) In-Process & Other Non-Indexed Citations and Ovid MEDLINE(R) <1946 to Present>
**Date of search:**	4.3.2013
**Search history:**	1 exp Patient Discharge/ (17145)<br>2 exp Patient Transfer/ (5333)<br>3 "Continuity of Patient Care"/ (13326)<br>4 (hospital adj3 discharge).mp. or (patient* adj3 (transition or transfer or discharge)).tw. [mp=title, abstract,<br>original title, name of substance word, subject heading word, keyword heading word, protocol supplementary concept, rare<br>disease supplementary concept, unique identifier] (32103)<br>5 1 or 2 or 3 or 4 (59623)<br>6 limit 5 to "all aged (65 and over)" (19465)<br>7 exp Patient Satisfaction/ (54960)<br>8 (patient adj2 (perspective or experience*)).tw. (11214)<br>9 7 or 8 (65287)<br>10 6 and 9 (803)<br>11 Primary Health Care/ (49380)<br>12 exp Community Health Services/ (462975)<br>13 (primary adj2 (care or "health care" or healthcare)).tw. (77030)<br>14 (("home based" or "homebased" or "home-based" or home or short term or short-term or shortterm) adj2 care).tw.<br>(17829)<br>15 exp Nursing Homes/ (30455)<br>16 "nursing home*".tw. (20439)<br>17 11 or 12 or 13 or 14 or 15 or 16 (575379)<br>18 10 and 17 (337)<br>19 ("early support* discharge" or "early discharge").tw. (1808)<br>20 18 not 19 (317)<br>21 Follow-Up Studies/ (459393)<br>22 20 not 21 (283)<br>23 limit 22 to "therapy (maximizes specificity)" (35)<br>24 limit 22 to "therapy (best balance of sensitivity and specificity)" (37)<br>25 22 not 24 (246)<br>26 therapy.fs. (1336566)<br>27 25 not 26 (207)
**Number of matches:**	207
**Comments:**	Stored in Reginas'account 2013-03-04-transition-gull-Medline

**Database/resource:**	Embase <1974 to 2013 March 01>
**Date of search:**	4.3.2013
**Search history:**	1 hospital discharge/ (55224)<br>2 "continuity of patient care".tw. (219)<br>3 ((hospital adj3 discharge) or (patient* adj3 (transition or transfer or discharge))).tw. (44618)

	4 1 or 2 or 3 (83320)<br>5 limit 4 to aged <65+ years> (24812)<br>6 patient satisfaction/ (77798)<br>7 (patient adj2 (perspective or experience*)).tw. (15485)<br>8 6 or 7 (92304)<br>9 5 and 8 (833)<br>10 primary health care/ (44446)<br>11 exp community care/ (92684)<br>12 (primary adj2 (care or "health care" or healthcare)).tw. (96273)<br>13 exp home care/ (50693)<br>14 (("home based" or "homebased" or "home-based" or home or short term or short-term or shortterm) adj2 care).tw.<br>(20476)<br>15 nursing home/ (39286)<br>16 "nursing home*".tw. (25375)<br>17 10 or 11 or 12 or 13 or 14 or 15 or 16 (291075)<br>18 9 and 17 (164)<br>19 ("early support* discharge" or "early discharge").tw. (2427)<br>20 18 not 19 (144)<br>21 follow up/ (687256)<br>22 20 not 21 (127)<br>23 limit 22 to "therapy (maximizes specificity)" (2)<br>24 limit 22 to "therapy (best balance of sensitivity and specificity)" (33)<br>25 22 not 24 (94)<br>26 th.fs. (1277512)<br>27 (25 not th).fs. (85)<br>28 from 27 keep 1-85 (85)<br><br>29 exp clinical handover/ (65)
**Number of matches:**	85 treff after duplicate control with Medline (207) in EndNote there are 49 new from from Embase<br>65 matches to term «clinical handover», sent sepaeratly by email.
**Comments:**	Stored in Reginas account 2013-03-04-transition-gull-EMBASE

**Database / resource:**	CINAHL (Ebsco) 1981 - present
**Date of search:**	19.3.2013
**Search history:**	S30 S18 AND S29 - 0<br>S29 S28 NOT S22 NOT S23 - 269<br>S28 (MH "Hand Off (Patient Safety)") - 275<br>S27 S22 NOT S23 NOT S26 - 241<br>S26 S24 Limiters - Clinical Queries: Therapy - Best Balance - 56<br>S25 S24 Limiters - Clinical Queries: Therapy - High Specificity - 16<br>S24 S22 NOT S23 - 297<br>S23 (MH "Prospective Studies+") - 142,010<br>S22 S18 NOT S21 - 357<br>S21 S19 OR S20 - 1,063<br>S20 TI ("early support* discharge" or "early discharge") OR AB ("early

	support* discharge" or "early discharge") - 630<br>S19 (MH "Early Patient Discharge") - 685<br>S18 S10 AND S17 - 386<br>S17 S11 OR S12 OR S13 OR S14 OR S15 OR S16 - 287,915<br>S16 TI "nursing home*" OR AB "nursing home*" - 12,211<br>S15 (MH "Nursing Homes+") - 15,672<br>S14 TI (("home based" or "homebased" or "home-based" or home or short term or short-term or shortterm) AND care) OR AB (("home based" or "homebased" or "home-based" or home or short term or short-term or shortterm) AND care) - 29,568<br>S13 TI (primary AND (care or "health care" or healthcare)) OR AB (primary AND (care or "health care" or healthcare)) - 43,002<br>S12 (MH "Community Health Services+") - 214,831<br>S11 (MH "Primary Health Care") - 26,830<br>S10 S6 AND S9 - 921<br>S9 (S7 OR S8) - 45,712<br>S8 TI (patient AND (perspective or experience*)) OR AB (patient AND (perspective or experience*)) - 25,035<br>S7 (MH "Patient Satisfaction") - 22,372<br>S6 S1 OR S2 OR S3 OR S4 Limiters - Age Groups: Aged: 65+ years - 9,618<br>S5 S1 OR S2 OR S3 OR S4 - 36,233<br>S4 TI (patient* AND (transition or transfer or discharge)) OR AB (patient* AND (transition or transfer or discharge)) - 19,185<br>S3 TI (hospital AND discharge) OR AB (hospital AND discharge) - 10,292<br>S2 (MH "Continuity of Patient Care+") - 8,893<br>S1 (MH "Patient Discharge+") - 14,055
**Number of matches:**	241, (after duplicate control with Medline and Embase i EndNote there is 205 new from Cinahl)
**Comments:**	Subject term «Home health care» is coverd under «Community health services»<br>Subject term “Hand off (Patient safety)” gave 275 matches. The term covers, unlike the corresponding subject terms in Medline og Embase, here both intra- og inter-handover, and also change over (change of guard). We therefore combined the term with S18, but it resulted in 0 treff.<br>MH = emneord (subject term), TI = Tittelord (title word), AB = ord i abstract (word in abstract)

**Database/resource:**	Svemed+
**Date of search:**	26.3.2013
**Search history:**	1 exp:"Patient Discharge" 448<br>2 exp:"Patient Transfer" 111<br>3 "Continuity of Patient Care" 0<br>4 "hospital discharge" OR "patient* transition" OR "patient* transfer" OR "patient* discharge" 546<br>5 #1 OR #2 OR #3 OR #4 546

	6 (#1 OR #2 OR #3 OR #4) AND exp:"Aged" 202<br>7 exp:"Patient Satisfaction" 1347<br>8 "patient* perspective" OR "patient* experience*" 213<br>9 #7 OR #8 1475<br>10 #6 AND #9 23
**Number of matches:**	23 matches. After removing duplets in EndNote (Cinahl, Medline), there were 21 treff from Svemed+
**Comments:**	Boolsk operator ADJ can not be used in Svemed+, therefor are some of the er noen av free text searches converted to phrase searches.

**Database/resource:**	Ovid PsycINFO <1806 to March Week 3 2013>
**Date of search:**	26.3.2013
**Search history**	1 hospital discharge/ or client transfer/ or discharge planning/ (1709)<br>2 exp "Continuum of Care"/ (883)<br>3 ((hospital adj3 discharge) or (patient* adj3 (transition or transfer or discharge))).tw. (4741)<br>4 1 or 2 or 3 (6279)<br>5 limit 4 to "380 aged <age 65 yrs and older>" (1485)<br>6 client satisfaction/ (3724)<br>7 (patient adj2 (perspective or experience*)).tw. (2416)<br>8 6 or 7 (6056)<br>9 5 and 8 (50)<br>10 primary health care/ (11327)<br>11 exp community services/ (23609)<br>12 (primary adj2 (care or "health care" or healthcare)).tw. (20933)<br>13 home care/ (4167)<br>14 (("home based" or "homebased" or "home-based" or home or short term or<br>short-term or shortterm) adj2 care).tw. (5925)<br>15 nursing homes/ (6221)<br>16 "nursing home*".tw. (8614)<br>17 10 or 11 or 12 or 13 or 14 or 15 or 16 (59661)<br>18 9 and 17 (13)
**Number of matches:**	Search 9: 50 matches (after removing duplets in EndNote: 38 matches)<br>Search 18: 13 matches (after removing duplets in EndNote 7 matches)
**Comments:**	No subject term for patient handoff/handover<br><br>Stored in search in Reginas account: 2013-03-26-transition-PsycINFO

Prisma Flow Diagram

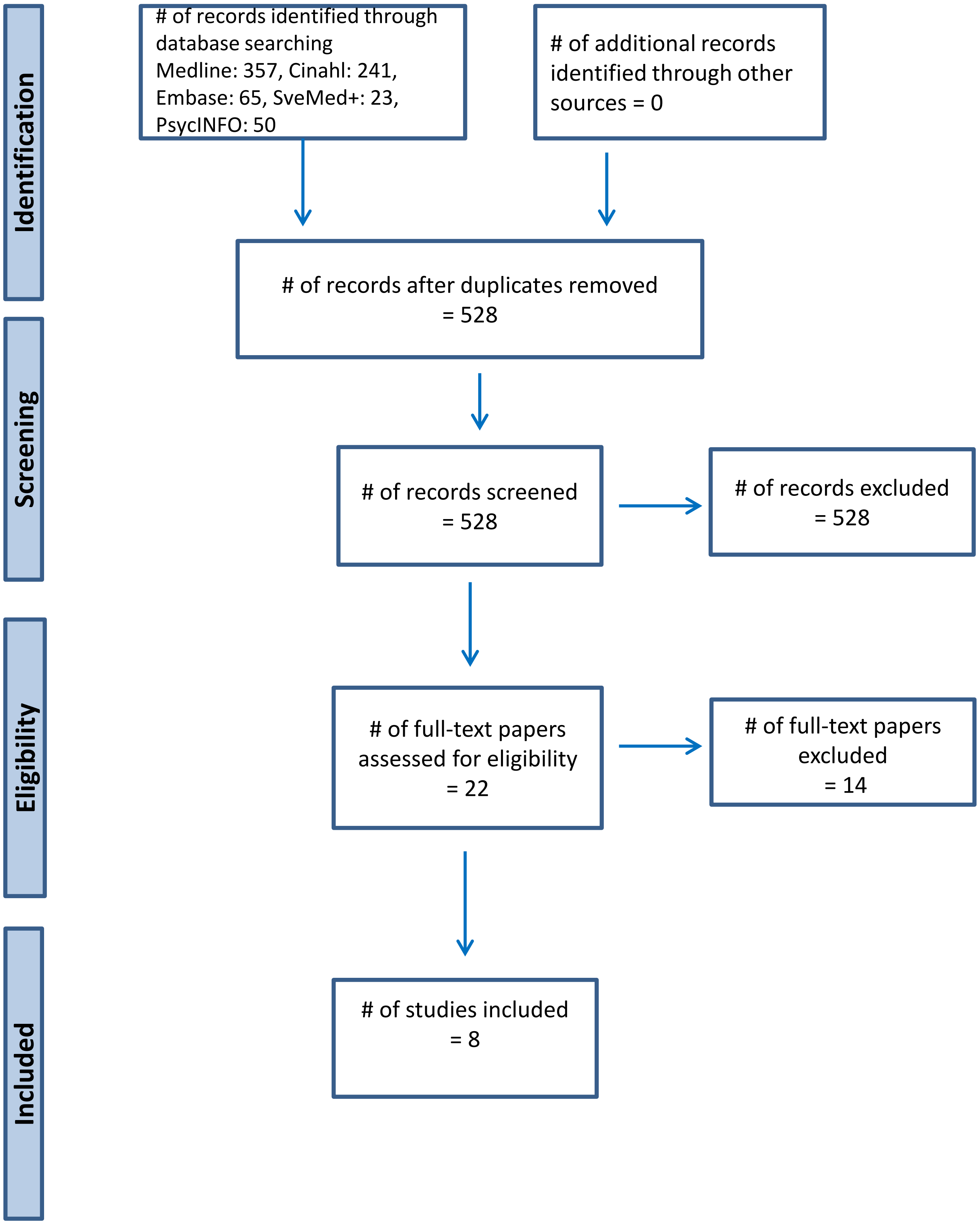

Objective: Patient experience on transition from hospital to primary health care. Results from a systematic literature search between 20.02.13-26.03.13

Author (year), link	Title	Design	Selection	Outcome	Results	Quality	Complete refererence, journal, link	Ethics
MEDLINE								
1.Perry, MAC (2011): http://www.sciencedirect.com/science/article/pii/S0738399 11000546X'	If I didn't have anybody, what would I have done?: Experiences of older adults and their discharge home after lower limb orthopaedic surgery.	Qualitative interviwes, analysed using interpretative phenomenological analysis (IPA).	11 patients (eight women) 65+ years were conducted between 6 and 12 weeks after discharge home from hospital	Patient experience	(i) Lack of a shared decision on when to go home; (ii)Dependent on family to go home and to feel confident there; and (iii) Trial and error rehabilitation.A further theme: a paternalistic medical model was also identified in participants' experiences of contact with health professionals.	Sample not randomized. One researcher interviewed all participants. Dropouts accounted.	Perry M.A.C, Hudson, S., ArdisK., Journal of Rehabilitation Medicine 2011; 43: 916–922http://www.sciencedirect.com/science/article/pii/S073839911000546X'	Approved by the Central Region Ethics Committee of New Zealand (CEN/09/07/047).
2.Foss C. (2011) :http://www.sciencedirect.com/science/article/pii/S073839 911000546X'	Elderly persons' experiences of participation in hospital discharge process.	Quantitative design. Questionnaire (76 questions) developed by the research team. Data collection October 2007-May 2009	254 patients (61,5% - 413 met the inclusion criteria) aged 80+ (mean=85 years)admitted to 14 hospitals and dicharged to 67 different municipalities. Recruited by the charge nurses in the 67 municipalites. Face-to-face interviewing during the 2-3 three weeks (mean 19.2 days) following patients discharge from hospital.	Patient experience	Patients expressed a clear preference for participation. No significant correlation between patients' wish for participation and experienced opportunity to share decisions.The chance to participate, whereas sociodemographic factors did not significantly affect on the likelihood discharge process. 65% of the patients stated that they felt a match between their own concerns and the concerns of the hospital professionals	Questionanaire not validated. A lot of interviewers involved, geriatric nurses or geriatric nurse students. Six-hours training course to educate interviewers.Dropouts accounted.	Foss C. Hofoss D. Patient Education and Counseling. 85(1):68-73, 2011 Oct http://www.sciencedirect.com/science/article/pii/S073839911000546X'	Approved by the East Norway Regional Ethics Committee for Medical Research and all municipalities involved.
3.Arora VM (2010) http://www.ncbi.nlm.nih.gov/pmc/articles/PMC3186075/pdf/nihms324166.pdf	Problems after discharge and understanding of communication with their primary care physicians among hospitalized seniors: a mixed methods study	Prospective mixed methods study. Datacollection February 2008 - July 2008	64 patients (56%,-114 met the inclusion criteria, mean age: 73 years) Inclusion:17 or below on Mini-Mental Status (22-point instrument),Inpatient interview within 48 hours after hospitalization and two weeks post-discharge phone interview (patients and/or proxys) - 12 open ended	Patient experience	Forty-two percent (27) of patients reported 42 different post-discharge problems. The most frequently reported problems were difficulty with follow-up appointments or tests (12).Other reported problems included readmission and return to the Emergency Department (10),problems with medications (8), not-prepared for discharge (8), and hospital complications orquestions (4). Thirty percent of PCPs were unaware of patient hospitalization. Patients were twice as likely to report a problem if their primary care physicians (PCP) was unaware of the hospitalization.The study suggests that many frail older patients reported problems after discharge and were twice as likely to do so when the patient's PCP was not aware of the hospitalization.	Dropouts not accounted	Arora VM. Prochaska ML. Farnan JM. D'Arcy MJ 5th. Schwanz KJ. Vinci LM. Davis AM. Meltzer DO. Johnson JK. Journal of Hospital Medicine (Online). 5(7):385-91, 2010 Sep. http://www.ncbi.nlm.nih.gov/pmc/articles/PMC3186075/pdf/nihms324166.pdf	No information
4. Wressle, E. (2006) http://onlinelibrary.wiley.com/doi/10.1111/j.1471-6712.2006.00390.x/pdf	Patient perspective on quality of geriatric care and rehabilitation – development and psychometri testing of a questionnaire	Qualitaive and quantitative design. Developing and testing a questionnaire in four phases..	Qualitative interviews 12 men and women (mean age 78 years) questionnaire 221 patients (89,1 %-248 met the inclusion criteria). Phoneinterview after discharge.	Patient experience, testing an validation of a questionairre	Qualitative interviews, findings: Information, respect of dignity, active participation, safety,interventions,physical environment an discharge planning. Questionnaire, results:The two highest scored items concerned feelings of safety and respect, while the lowest scored item was staff exposure to stress. Eighty-eight per cent of the participants scored 'Totally agree' on the global item that concerned satisfaction with care.	Validated instrument. Cronbach alpha 0.79. Dropouts accounted, mean age 79 years, ranging between 50 and 97. The interviewers were seven nurses, one assistant nurse and two medical social workers. Geriatric care and rehabilitation (Kalmar hospital, Sverige)	Wressle E. Eriksson L. Fahlander A. Rasmusson IM. Tedemalm U. Tangmark K. Scandinavian Journal of Caring Scandinavian Journal of Caring Sciences. 20(2):135-42, 2006 Jun. Sciences. 20(2):135-42, 2006 Jun. http://onlinelibrary.wiley.com/doi/10.1111/j.1471-6712.2006.00390.x/pdf	Approved by the Ethics Committee in Faculty of Health Sciences in Linko¨ping.

5.Tyson, S. http://cre.sagepub.com/content/14/4/381.full.pdf+html	Discharge and follow-up for people with stroke:what happens and why	Quantiative design. Part 1.A criterion-based process audit. Part 2. Surveys.	Documented notes of 98 stroke patients admitted and discharged over a four-month period. Patient satisfaction survey: 93 surviving stroke patients. Staff opinion survey: general practitioners, hospital doctors, therapists and nurses treating stroke patients throughout the district.	Patients' satisfaction and staff opinion of the service.	A poor level of service was found. Patients were dissatisfied with the information, support services and therapy they received. The main reasons for the shortcomings were lack of awareness of the services provided, professionals' low expectations of patients' abilities, and limitations of community-based therapy services.	Questinnaire not validatet. Dropouts not accounted.	Tyson S. Turner G. Clinical Rehabilitation. 14(4):381-92, 2000 Aug. Clinical Rehabilitation. 14(4):381-92, 2000 Aug. http://cre.sagepub.com/content/14/4/381.full.pdf+html	No information
6. Fairhurst K. (1996) http://intqhc.oxfordjournals.org/content/8/2/167.full.pdf	*The quality of hospital discharge: a survey of discharge arrangements for the over-65s.*	Quantitative design. A three-phase study comprising semistructured interviews with patients and or their carers, follow-up postal questionnaires, and a postal survey of the views of proffesionals involved in the discharge.	Patients 65 years and over discharged from four hospitals,	Patient and/or carers, and health professionals satisfaction on discharge arrangement	138 patients were intervied, 128 participated in the survey (replies 86%). Dropouts accounted. A large majority of patients (80%) felt they had been adequately consulted about arrangements for their discharge, but less than 2% of health and social care professionals considered all discharges satisfactory. This apparent disparity between patient and professional dews may be explained by low levels of expectation among patients in this aspect of their care and their reluctance to express views which they fear may compromise future care. 13 (12%) felt they had been sent home too soon.	Questionanaire not validated. High responsrate. Dropouts accounted.	Fairhurst K. Blair M. Cutting J. Featherstone M. Hayes B. Howarth M. Rose D. Stanley I. International Journal for Quality in Health Care. 8(2):167-74, 1996 Apr.http://intqhc.oxfordjournals.org/content/8/2/167.full.pdf	No information found
**EMBASE**								
7.Jones, D. (1994) http://ageing.oxfordjournals.org/content/23/2/91.long	Hospital Care and Discharge: Patients' and Carers' Opinions	Quantitative design. 12-page questionnaire, with a 4-page questionnaire enclosed to pass to the person who had given most help in the 4 weeks immediately following return from hospital.	Questionnaires were sent to 1084 patients three months after discharge.	Patients and cares opinion of hospital care and discharge planning	960 questionnaires were completed, a response rate of 89%. Carers were more likely than patients to be dissatisfied with discharge arrangements, including length of notice, and more likely to think that discharge had been premature. 38% (patients) did not remember discussing discharge with hospital staff	Questionanaire not validated. Large n, high responsrate. Dropouts not accounted. Very comprehensive questionnaire.	Jones, D. and C. Lester, Age and Ageing, 1994. 23(2): p. 91-96. http://ageing.oxfordjournals.org/content/23/2/91.long	No information found
8.Bisset, A.F (1997) http://www.ncbi.nlm.nih.gov/pmc/articles/PMC1410073/pdf/brjgenprac00091-0009.pdf	Stroke services, in general practice are they satisfactory?	Quantiative design.	Data obtained on 150 patients 6 weeks after discharged to home or nursing home.Patients were blindly randomized to two groups: 75% to receive postal questionnaires and 25% to receive a visit by an audit assistant who administered the same [illegible]	To determine general practitioners' (GPs') and their patients' satisfaction with hospital and community [illegible]	Rsponserate 91%, mean age 72 years.Patients were generally satisfied with services. 83 patients felt that things had been well prepared for their return home. 14 patients stated that they had wanted but not received particular services.	Dropouts accounted. Questionanaire not validated.Moderate n, dropouts accounted. Blindly randomized.	Soderback, I., Occupational Therapy International, 2008. 15(1): p. 18-31. http://onlinelibrary.wiley.com/doi/10.1002/oti.241/pdf	No information found
