# Supplementary material for: Elderly patients’ (≥65 years) experiences associated with discharge; Development, validity and reliability of the Discharge Care Experiences Survey
Source: PLoS One. 2018 Nov 7;13(11):e0206904. doi: 10.1371/journal.pone.0206904 (PMC6221326; doi:10.1371/journal.pone.0206904)
Supplement: S3 File — Erfaringer knyttet til utskriving og tiden etter sykehusoppholdet. (PDF) [file pone.0206904.s003.pdf]

Erfaringer knyttet til utskriving og tiden etter sykehusoppholdet

Vi ønsker å vite mer om hva pasienter erfarer i forbindelse med utskriving og tiden etter sykehusoppholdet. Målsettingen er å forbedre kvaliteten på tilbudet pasienter får. Alle svaralternativene kan benyttes, men *sett kun ett kryss på hvert spørsmål*.

	Ikke i det hele tatt	I liten grad	I noen grad	I stor grad	I svært stor grad	Ikke aktuelt
Erfaringer knyttet til utskriving:						
1. I forbindelse med utskrivingen fikk jeg fortalt sykehuspersonalet hva jeg selv mente var viktig	☐	☐	☐	☐	☐	☐
2. Da sykehuspersonalet vurderte hvilke helsetjenester jeg kom til å ha behov for etter sykehusoppholdet, tok de hensyn til hva jeg og mine pårørende ønsket	☐	☐	☐	☐	☐	☐
3. Ved utskriving fra sykehuset hadde jeg god forståelse for hva som var mitt ansvar for å ta vare på egen helse	☐	☐	☐	☐	☐	☐
4. Ved utskriving fra sykehuset forsto jeg klart hensikten med å ta de forskjellige medisinene mine	☐	☐	☐	☐	☐	☐
5. Det var viktig for meg å ha innflytelse på når jeg skulle bli utskrevet fra sykehuset	☐	☐	☐	☐	☐	☐
6. Jeg opplevde å bli for tidlig utskrevet	☐	☐	☐	☐	☐	☐
Erfaringer knyttet til tiden etter sykehusoppholdet:						
7. Jeg har hatt problemer med å forstå instruksjonene jeg fikk ved utskriving fra sykehuset	☐	☐	☐	☐	☐	☐
8. Jeg har hatt problemer med å følge instruksjonene jeg fikk ved utskriving fra sykehuset	☐	☐	☐	☐	☐	☐
9. Jeg har hatt problemer med å få hjelp fra min fastlege	☐	☐	☐	☐	☐	☐
10. Jeg har hatt problemer med å ta medisinene mine	☐	☐	☐	☐	☐	☐
11. Jeg har hatt problemer med å få tak i medisiner	☐	☐	☐	☐	☐	☐
12. Jeg har hatt problemer med å få i meg tilstrekkelig næring	☐	☐	☐	☐	☐	☐
13. Jeg har hatt problemer med å utføre daglige aktiviteter (f.eks. personlig hygiene, påkledning eller matlaging)	☐	☐	☐	☐	☐	☐
14. Jeg har vært stresset	☐	☐	☐	☐	☐	☐
15. Jeg har vært deprimert	☐	☐	☐	☐	☐	☐
16. Jeg skulle gjerne hatt flere personer å snakke med, og støtte meg til, etter at jeg ble utskrevet fra sykehuset	☐	☐	☐	☐	☐	☐
